# A new method for assessment of sediment-associated contamination risks using multivariate statistical approach

**DOI:** 10.1016/j.mex.2018.03.005

**Published:** 2018-03-30

**Authors:** Nsikak U. Benson, Adebusayo E. Adedapo, Omowunmi H. Fred-Ahmadu, Akan B. Williams, Essien D. Udosen, Olusegun O. Ayejuyo, Abass A. Olajire

**Affiliations:** a Analytical and Environmental Chemistry Unit, Department of Chemistry, Covenant University, Ota, Nigeria; b Department of Chemistry, University of Uyo, Akwa Ibom State, Nigeria; c Department of Chemistry, University of Lagos, Lagos, Nigeria; d Industrial and Environmental Chemistry Unit, Department of Pure and Applied Chemistry, Ladoke Akintola University of Technology, Ogbomoso, Nigeria

**Keywords:** New ecological risk indices, Fractionation, Heavy metals, Sediment pollution, Contamination indices, Principal component analysis

## Abstract

This paper presents the assimilation of heavy metal concentration data from sequential extraction method (SEM) with metal toxicity factors to develop and propose two new sediment quality indices modified hazard quotient (*m*HQ) and ecological contamination index (ECI), to predict the potential ecological risks associated with sediment contamination. Chemical speciation data of five heavy metals: cadmium (Cd), chromium (Cr), copper (Cu), nickel (Ni), and lead (Pb) from five coastal aquatic ecosystems of the Equatorial Atlantic Ocean were used in the assessment of the degree of heavy metal contamination. Evaluation based on ECI indicated that sediments of most aquatic ecosystems were considerably to highly contaminated. The results showed that the proposed indices are reliable, precise, and in good agreement with similar existing indices used for evaluating the severity of sediment-associated contamination by heavy metals. The principal component analysis (PCA) and factor analysis indicated that heavy metals in the benthic sediments were mostly from anthropogenic sources.

• New indices – modified hazard quotient (*m*HQ) and ecological contamination index (ECI) - were developed for predicting sediment-associated risk adverse effects.
• Newly proposed indices agree closely with the existing pollution indices.
• Pollution indices reveal significant anthropogenic contamination by Cd and Pb.

## Graphical abstract

**Figure:**
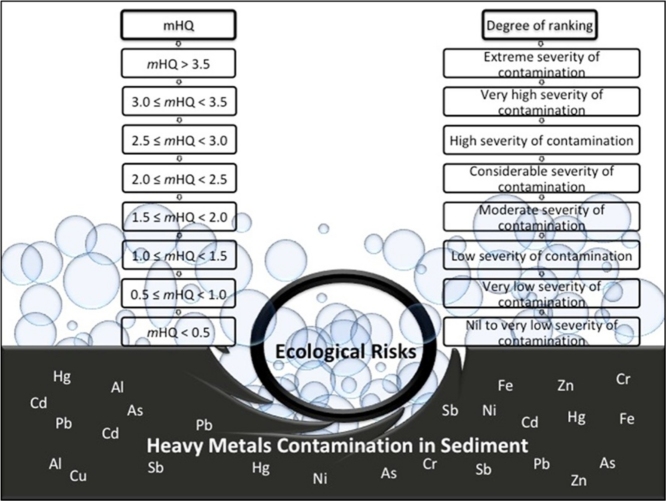

**Specifications Table**

**Table:** 

Subject area	*Environmental Science*
More specific subject area	*Analytical Chemistry*
Method name	*New ecological risk indices*

## Method details

### Background

Sediments are integrated components of aquatic ecosystems, and have been recognized as sinks of heavy metals [[1], [2], [3], [4], [5], [6], [7]]. Heavy metal concentration data are commonly applied in monitoring and assessing the degree of contamination of aquatic environments using sediment quality indices [2,[8], [9], [10], [11], [12], [13], [14]]. Reports indicate that heavy metals in sediments could pose considerable adverse effects on aquatic animals, plants and the environment due to their bioaccumulation potential, non-biodegradability, and toxicity [4,[15], [16], [17], [18], [19], [20], [21]]. Several empirical and statistical indices have also been developed as contamination assessment tools for monitoring sediments in aquatic ecosystems. Sediment quality indices have been developed and widely applied in assessment of heavy metal contamination in aquatic ecosystems including risk assessment code [22], ecological risk index [23], pollution load index [24], modified degree of contamination [25], modified risk assessment code [26], and contamination severity index [7]. Although these approaches have existed since the early 80′s and are widely accepted and employed in sediments associated studies, each of these indices and reference values has their peculiar reliability advantages and limitations.

In this study, two new composite indices, namely, modified hazard quotient (*m*HQ) and ecological contamination index (ECI) have been developed, proposed, and applied as new sediment quality assessment tools, based on the assimilation of heavy metal concentration data from sequential extraction method with metal toxicity factors to assess potential degree of metal contamination in sediments from multiple tropical estuaries and freshwater ecosystems off the Equatorial Atlantic Ocean. The report provides a better understanding of the metal pollution status in the aquatic ecosystems.

## Materials and method

### Study Area and sampling

Details of the sampling area, sampling technique and extraction procedure, heavy metals instrumental and data analysis have been previously reported [2,10]. Five mesotidal and intertidal coastal water systems were considered. The aquatic ecosystems include Douglas Creek (DOU), Okorotip Creek (OKT), Stubbs Creek (STB), Qua Iboe Estuary (QUE) and Qua Iboe River (QUR). Sampling sites within the water bodies of these ecosystems were clearly mapped and designated for the collection of benthic sediments during the wet (June–August) and dry (November–January) seasons of the year. Benthic sediment samples from each ecosystem were collected using a modified van Veen (0.1 m^2^) grab sampler and were preserved in clean, well-labelled glass bottles. After collection, the samples were all stored in ice-packed coolers and transported to the laboratory. These samples were further refrigerated in the laboratory at 4 °C to inactivate microbes and to preserve the integrity of the samples prior to analysis. In total, ninety (90) benthic sediment samples were collected from designated study locations. In the laboratory, the sediment samples were dried in an oven maintained at 105 ± 0.5 °C, homogenized, comminuted using a hand mortar and sieved using a 2 mm mesh sieve prior to leaching. Coning and quartering methods were used to obtain subsamples from the respective composite samples.

### Sample extraction, instrumentation and data analysis

**Fig. 1:**
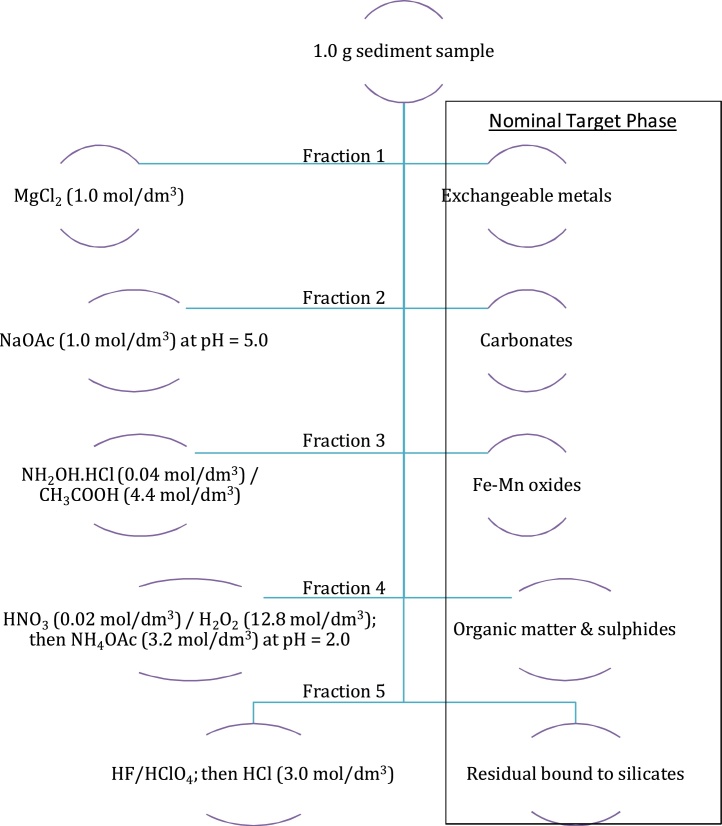
Flowchart showing the sequential procedure for chemical fractionation.

The Tessier’s procedure (Fig. 1) designed to separate heavy metals into five operationally defined fractions: exchangeable (F1), carbonate bound (F2), Fe-Mn oxides bound (F3), organic bound (F4) and residual fractions (F5) was used for this study [2]. The determinations of cadmium (Cd), copper (Cu), chromium (Cr), lead (Pb) and nickel (Ni) were performed using inductively coupled plasma spectrophotometer (ICP-AES). The detection limits were 0.02, 0.01, 0.02, 0.02 and 0.01 mg/kg for Cd, Cr, Cu, Pb and Ni, respectively. Data analyses were carried out with XLSTAT-Pro software (AddinSoft Inc. USA). The monthly fractionation concentrations (F1 + F2 + F3 + F4 + F5) of heavy metals (Cd, Cr, Cu, Ni, and Pb) in benthic sediments from the investigated aquatic ecosystems are presented in Table 1.

**Table 1:** Monthly concentration (mean ± s.d, mg/kg) of trace metals in studied aquatic ecosystems.

		QUE	DOU	STB	OKT	QUR
Coordinates		4.53 °S, 7.99 °N	4.55 °S, 8.00 °N	4.60 °S, 7.99 °N	4.56 °S, 7.93 °N	4.58 °S, 7.93 °N
Cadmium	Jun	4.38 ± 1.19	4.88 ± 1.31	5.02 ± 1.35	4.47 ± 1.13	5.01 ± 1.35
	Jul	4.96 ± 1.41	4.63 ± 1.22	5.08 ± 1.34	5.67 ± 1.78	5.63 ± 1.67
	Aug	4.71 ± 1.27	4.52 ± 1.25	4.99 ± 1.36	4.86 ± 1.27	4.59 ± 1.23
	Nov	4.84 ± 1.33	5.21 ± 1.44	4.47 ± 1.16	4.41 ± 1.55	4.89 ± 1.38
	Dec	4.71 ± 1.25	4.80 ± 1.27	4.41 ± 1.17	4.71 ± 1.26	4.69 ± 1.26
	Jan	4.64 ± 1.26	4.78 ± 1.23	4.33 ± 1.12	4.67 ± 1.26	4.64 ± 1.24

Chromium	Jun	20.37 ± 4.09	19.02 ± 3.63	20.34 ± 3.98	21.51 ± 4.29	11.12 ± 1.81
	Jul	20.08 ± 4.04	20.63 ± 3.92	19.86 ± 3.70	20.84 ± 4.19	18.11 ± 3.55
	Aug	18.93 ± 3.69	17.50 ± 3.22	20.37 ± 4.02	20.93 ± 4.21	15.16 ± 2.59
	Nov	20.60 ± 4.17	18.95 ± 3.55	19.05 ± 3.62	19.54 ± 3.83	17.09 ± 3.39
	Dec	18.61 ± 3.63	19.90 ± 3.88	20.73 ± 4.07	18.44 ± 3.34	28.52 ± 7.21
	Jan	20.52 ± 3.99	20.11 ± 3.88	18.78 ± 3.58	20.06 ± 3.73	18.37 ± 3.46

Copper	Jun	31.74 ± 4.80	40.70 ± 7.35	43.01 ± 8.08	30.86 ± 4.53	43.73 ± 8.95
	Jul	36.43 ± 5.84	38.61 ± 6.62	39.86 ± 6.94	40.69 ± 7.33	35.07 ± 5.65
	Aug	38.73 ± 6.69	36.39 ± 6.16	43.08 ± 7.93	30.26 ± 4.97	38.56 ± 6.67
	Nov	31.05 ± 4.58	39.31 ± 6.86	40.54 ± 6.99	39.57 ± 7.39	41.01 ± 7.44
	Dec	35.75 ± 5.68	37.25 ± 6.41	38.00 ± 6.54	42.02 ± 7.61	39.87 ± 7.43
	Jan	38.29 ± 6.25	36.55 ± 6.09	37.43 ± 6.31	41.49 ± 7.48	39.26 ± 6.68

Lead	Jun	177.63 ± 4.95	166.42 ± 9.94	181.48 ± 7.24	183.48 ± 8.79	162.00 ± 8.54
	Jul	180.03 ± 4.23	172.50 ± 2.91	187.06 ± 8.08	167.61 ± 0.87	182.37 ± 6.05
	Aug	231.52 ± 6.82	177.80 ± 3.59	175.37 ± 6.90	190.37 ± 7.83	173.49 ± 3.95
	Nov	185.81 ± 8.10	185.11 ± 6.68	176.86 ± 5.30	169.25 ± 3.78	178.42 ± 4.12
	Dec	186.48 ± 8.00	181.59 ± 6.36	180.21 ± 7.12	171.71 ± 8.64	175.72 ± 5.13
	Jan	185.07 ± 6.59	186.58 ± 8.71	183.34 ± 5.51	185.38 ± 6.06	183.13 ± 6.51

Nickel	Jun	2.06 ± 0.35	2.24 ± 0.39	2.23 ± 0.39	2.05 ± 0.36	2.17 ± 0.39
	Jul	2.60 ± 0.59	2.12 ± 0.34	2.25 ± 0.40	2.17 ± 0.39	2.23 ± 0.41
	Aug	2.17 ± 0.38	2.13 ± 0.34	2.19 ± 0.36	2.20 ± 0.39	2.03 ± 0.41
	Nov	2.25 ± 0.40	2.17 ± 0.37	2.23 ± 0.41	2.26 ± 0.39	2.28 ± 0.40
	Dec	2.27 ± 0.43	2.25 ± 0.39	2.13 ± 0.33	2.09 ± 0.40	2.16 ± 0.41
	Jan	2.26 ± 0.41	2.27 ± 0.40	2.18 ± 0.36	2.22 ± 0.38	2.26 ± 0.40

(DOU = Douglas creek; OKT = Okorotip creek; STB = Stubbs creek; QUE = Qua Iboe estuary; QUR = Qua Iboe river).

## Principal component analysis

The rotated factor loadings of principal component analysis (PCA) were used to evaluate the interrelationships of trace metals in benthic sediments from the five studied aquatic ecosystems as given in Table 2. The different trace metals contamination behaviours were observed in all the five studied ecosystems. As shown in Table 2, there were two principal components (PC1 and PC2) for sedimentary heavy metals at the DOU, OKT, STB, QUE and QUR sites. Multivariate statistical analyses using PCA showed that heavy metals pollution in these ecosystems originated from two principal sources – anthropogenic and lithogenic sources. The 1st principal component (PC1) indicated heavy metal contamination from anthropogenic sources, while the second principal component (PC2) represented natural sources of contamination. Cd, Pb and Cu may have common human-induced sources such as industrial and vehicular related activities. More so, Cr and Ni indicate a mixed-type origin from natural rock weathering processes and anthropogenic on- and off-shore-based industrial related activities.

**Table 2:** Loadings of two principal components for benthic sediment variables.

	DOU		OKT		STB		QUR		QUE	
	PC1	PC2	PC1	PC2	PC1	PC2	PC1	PC2	PC1	PC2
Load of Cd	0.634	0.452	0.234	0.936	0.953	0.114	0.576	0.734	0.484	−0.758
Load of Cr	0.160	0.345	−0.786	0.508	0.439	−0.635	−0.682	0.459	0.485	0.708
Load of Cu	0.750	−0.144	0.943	−0.002	0.907	−0.252	0.821	−0.149	−0.832	−0.068
Load of Ni	0.125	0.558	0.368	−0.095	0.623	0.716	0.467	0.865	0.522	−0.431
Load of Pb	−0.401	0.587	−0.817	−0.265	−0.060	0.783	0.662	−0.590	0.913	0.210
Eigenvalue	1.705	1.601	2.366	1.214	2.317	1.605	2.128	1.868	2.268	1.311
Variability (%)	34.108	32.022	47.314	24.275	46.337	32.110	42.565	37.360	45.365	26.226
Cumulative %	34.108	66.130	47.314	71.589	46.337	78.447	42.565	79.925	45.365	71.591

## Newly developed contamination index

### Modified hazard quotient (mHQ)

In the present study, a new index for evaluating sediment pollution based on the degree of contamination by individual heavy metal is formulated and proposed. This new approach enables the assessment of contamination by comparing metal concentration in sediment with the synoptic adverse ecological effects distributions for slightly differing threshold levels (TEL, PEL and SEL) as earlier reported [27]. The determination of modified hazard quotient (*m*HQ) of metals is an important assessment tool that elucidates the degree of risk of each heavy metal to aquatic environment and the biota, and is computed using the following mathematical formula:

(1) mHQ=Ci1TELi+1PELi+1SELi12

where, *C_i_* is the measured concentration of heavy metal in the sediment samples, TEL*_i_*, PEL*_i_* and SEL*_i_* are acronym for the threshold effect level, probable effect level and severe effect level for *i*th metal respectively. In the equation, the square root is introduced as a drawdown function for mathematical and ranking considerations. The proposed classification of contamination by single metal using the newly developed index is presented in Table 3.

**Table 3:** Classification of Modified Hazard Quotient (*m*HQ).

*m*HQ	Degree of risk
*m*HQ > 3.5	Extreme severity of contamination
3.0 ≤ *m*HQ < 3.5	Very high severity of contamination
2.5 ≤ *m*HQ < 3.0	High severity of contamination
2.0 ≤ *m*HQ < 2.5	Considerable severity of contamination
1.5 ≤ *m*HQ < 2.0	Moderate severity of contamination
1.0 ≤ *m*HQ < 1.5	Low severity of contamination
0.5 ≤ *m*HQ < 1.0	Very low severity of contamination
*m*HQ < 0.5	Nil to very low severity of contamination

### Ecological contamination index (ECI)

In this study, we proposed a reliable index known as ecological contamination index (ECI) for an overall ecological risk assessment of sediment contamination by heavy metals. The ECI is an aggregative empirical approach that estimates the risks associated with an ecosystem using a source-specific factor derived primarily from principal component analysis/factor analysis. The proposed formula for ECI is mathematically expressed as:

(2) $$ECI=B_{n}\sum_{i=1}^{n}mHQ_{i}$$

where, *B_n_* = the reciprocal of the derived eigenvalue of heavy metal concentrations only. The proposed ranking of risks posed by heavy metals to ecological systems using the proposed index is given in Table 4.

**Table 4:** Classification of Ecological Contamination Index (ECI).

ECI	Degree of contamination
ECI > 7	Extremely contaminated
6 ≤ ECI < 7	Highly contaminated
5 ≤ ECI < 6	Considerably to highly contaminated
4 ≤ ECI < 5	Moderately to considerably contaminated
3 ≤ ECI < 4	Slightly to moderately contaminated
2 ≤ ECI < 3	Uncontaminated to slightly contaminated
ECI < 2	Uncontaminated

The calculated *m*HQs indicated that the severity of sediment-associated pollution of the five heavy metals were in the descending sequence of Cd > Pb > Cu > Cr > Ni. This trend is in good agreement with other contamination sequence obtained for pollution assessment indices earlier reported for these ecosystems [2,9,10] and other reports [12,28]. Results indicated that Cd recorded very high degree of contamination followed by Pb with severity ranking characterized by high degree of contamination. However, Cu, Cr and Ni generally showed low to very low degree of contamination during the wet and dry seasons at all the investigated sites.

The multi-elemental potential ECIs for all sites were 4.06, 3.80, 3.46, 5.06, and 3.73 for sites QUE, QUR, OKT, DOU, and STB, respectively. The calculated ECIs indicated that the ecosystems were characterized by a slightly contaminated to a highly contaminated degree of pollution. The ecological risk ranking based on percentage contribution to ECI followed the sequence Cd > Pb > Cu > Cr > Ni, while the severity of ecosystem pollution based on the five heavy metals decreased in the following sequence: DOU > QUE > QUR > STB > OKT. Again, Cd contributed significantly to the ecological contamination risk index of these ecosystems than other heavy metals. The reliability and accuracy of the newly proposed formulae for assessment of sediment-associated heavy metals in aquatic ecosystems were ascertained by comparison with other existing pollution indices. Results indicated that the ECI is a reliable and useful pollution tool that can be used to estimate the extent of pollution, site-specific status and aggregative contamination effects by heavy metals in aquatic ecosystems.

## Conclusion

Heavy metals levels and contamination status in benthic sediments of five equatorial estuarine and riverine ecosystems were evaluated using existing pollution indices. Newly proposed index was used to evaluate the holistic ecological severity risk of sediment-associated heavy metals. The ECI is an aggregative index that represents the overall contamination pedigree and associated ecological risks based on the contribution of all the heavy metals in an aquatic ecosystem. The risk assessment indices employed in the present study reveal significant contamination risk by Cd and Pb. The PCA revealed that both anthropogenic and lithogenic sources are responsible for the possible contamination of the investigated ecosystem by Cd, Cr, Cu, Ni and Pb. Estimation of potential risks by metals using the proposed ECI revealed possible pollution hotspot sites. A comparison of the newly proposed indices with existing pollution indices reveals very good agreement.

## Method validation

The reliability and accuracy of the newly proposed formulae for assessment of sediment-associated heavy metals in aquatic ecosystems were ascertained by detailed comparison of the severity rankings of new indices with existing pollution indices. The trends of sediment metal contamination using existing and newly proposed indices were consistent (Table 5). Results indicated that the *m*HQ and ECI were reliable and useful pollution tools with potential to estimate the degree of pollution, site-specific status and aggregative contamination effects by heavy metals in aquatic ecosystems.

**Table 5:** Comparison of contamination trends using existing and newly proposed pollution contamination indices.

Pollution index		Pollution sequence of heavy metals				Reference
	Douglas creek	Okorotip creek	Stubbs creek	Qua Iboe Estuary	Qua Iboe River	
CF	Cd > Pb > Cu > Cr > Ni	Cd > Pb > Cu > Cr > Ni	Cd > Pb > Cu > Cr > Ni	Cd > Pb > Cu > Cr > Ni	Cd > Pb > Cu > Cr > Ni	Benson et al. [10]
% DC	Cd > Pb > Cu > Cr > Ni	Cd > Pb > Cu > Cr > Ni	Cd > Pb > Cu > Cr > Ni	Cd > Pb > Cu > Cr > Ni	Cd > Pb > Cu > Cr > Ni	
PERI	Cd > Pb > Cu > Cr > Ni	Cd > Pb > Cu > Cr > Ni	Cd > Pb > Cu > Cr > Ni	Cd > Pb > Cu > Cr > Ni	Cd > Pb > Cu > Cr > Ni	
% Ri	Cd > Pb > Cu > Cr > Ni	Cd > Pb > Cu > Cr > Ni	Cd > Pb > Cu > Cr > Ni	Cd > Pb > Cu > Cr > Ni	Cd > Pb > Cu > Cr > Ni	
PCI	Cd > Pb > Cu > Cr > Ni	Cd > Pb > Cu > Cr > Ni	Cd > Pb > Cu > Cr > Ni	Cd > Pb > Cu > Cr > Ni	Cd > Pb > Cu > Cr > Ni	
ICF	Cu > Cr > Ni > Cd > Pb	Cu > Cr > Ni > Cd > Pb	Cu > Cr > Ni > Cd > Pb	Cu > Cr > Ni > Cd > Pb	Cu > Cr > Ni > Cd > Pb	Benson et al. [4]
CSI	Cd > Cr > Cu > Ni > Pb	Cd > Cr > Cu > Ni > Pb	Cd > Cr > Cu > Ni > Pb	Cd > Cr > Cu > Ni > Pb	Cd > Cr > Cu > Ni > Pb	Pejman et al. [7]
mRAC	Ni > Cd > Cr > Cu > Pb	Ni > Cd > Cr > Cu > Pb	Ni > Cd > Cr > Cu > Pb	Ni > Cd > Cr > Cu > Pb	Ni > Cd > Cr > Cu > Pb	Pejman et al. [7]
HQ	Cd > Pb > Cu > Cr > Ni	Cd > Pb > Cu > Cr > Ni	Cd > Pb > Cu > Cr > Ni	Cd > Pb > Cu > Cr > Ni	Cd > Pb > Cu > Cr > Ni	Benson et al. [2]
**mHQ**	Cd > Pb > Cu > Cr > Ni	Cd > Pb > Cu > Cr > Ni	Cd > Pb > Cu > Cr > Ni	Cd > Pb > Cu > Cr > Ni	Cd > Pb > Cu > Cr > Ni	New proposed formula
**ECI**	Cd > Pb > Cu > Cr > Ni	Cd > Pb > Cu > Cr > Ni	Cd > Pb > Cu > Cr > Ni	Cd > Pb > Cu > Cr > Ni	Cd > Pb > Cu > Cr > Ni	New proposed formula

CF = Contamination factor; %DC=% contribution of single metal to degree of contamination; PERI = Potential ecological risk index; %Ri=% contribution to risk index; PCI = Potential contamination index; ICF = Individual contamination factor; CSI = Contamination severity index; mRAC = Modified risk assessment code; HQ = Hazard quotient; mHQ= Modified hazard quotient; ECI= Ecological contamination index.
